# Effect of Peat on Physicomechanical Properties of Cemented Brick

**DOI:** 10.1155/2014/328516

**Published:** 2014-04-10

**Authors:** Syed Mofachirul Islam, Roslan Hashim, A. B. M. Saiful Islam, Ryan Kurnia

**Affiliations:** Department of Civil Engineering, Faculty of Engineering, University of Malaya, 50603 Kuala Lumpur, Malaysia

## Abstract

The popularity of low cost, lightweight, and environmentally affable masonry unit in building industry carries the need to investigate more flexible and adaptable brick component as well as to retain the requirements confirmed in building standards. In this study, potential use of local materials used as lightweight building materials in solving the economic problems of housing has been investigated. Experimental studies on peat added bricks have been carried out. It demonstrates the physicomechanical properties of bricks and investigates the influence of peat, sand, and cement solid bricks to the role of various types of constructional applications. The achieved compressive strength, spitting strength, flexural strength, unit weight, and ultrasonic pulse velocity are significantly reduced and the water absorption is increased with percentage wise replacement of peat as aggregate in the samples. The maximum 20% of (% mass) peat content meets the requirements of relevant well-known international standards. The experimental values illustrate that, the 44% volumetric replacement with peat did not exhibit any sudden brittle fracture even beyond the ultimate loads and a comparatively smooth surface is found. The application of peat as efficient brick substance shows a potential to be used for wall and a viable solution in the economic buildings design.

## 1. Introduction

Brick and block are a fundamental building material for the low-cost housing and the multistoried building construction. Housing construction using earth-based brick or block materials is economical for the majority of urban areas due to the energy saving in manufacturing compared to conventional bricks and the transport savings, which directly affect the total cost. Zami and Lee [1] in their publication stated, “when produced locally with natural resources, semiskilled labor, and few transport needs, contemporary earth construction for low-cost urban housing can be very cost effective according to context and available skills.” The most common building bricks are the traditional fired clay brick and a huge amount of energy is depleted throughout its production [2]. The trend is presently moving on the new schemes and products because the conventional brick can make a major contribution to track energy usage, climate change, and greenhouse gas emissions [3–5].

On the other hand, house construction by traditional bricks is too costly for the majority of urban areas due to transport costs, which directly has an effect on the total material cost. By utilizing local raw materials, it decreases the material cost because transportation is significantly reduced and making a much more affordable option for poor communities [6]. Berge [7] stated that the transportation energy of building materials plays an important role in its low environmental performance and the uses of local materials as it happens with earth base should be prioritized.

The uses of local materials in the building sector can contribute decisively to decrease in energy consumption. The engineers have been taken to convert the local materials to useful building and construction materials. Accumulation of raw materials of bricks is today's one of significant problems and environmental and cost concerns, especially in area such as peat region. Using of such peat soil as building materials appears to be viable solution not only to environment pollution but also to the problem of economical design of buildings. The increase in the popularity of using environmentally friendly low cost and lightweight construction materials in building industry brings the need for searching more innovative, flexible, and versatile composites. The most important aspects of innovation might be in the development of integrated local products.

Methods and data exist for dealing with the common building walls, but new systems and products are generally lacking such data. Only a few research works about the potential utilization strategies of peat in the building materials industry [8, 9] are existent in the literature. In their research, tests are carried out on the certain number of parameters. The use of peat soil in cement based brick materials focuses on the use of peat as an aggregate and evaluates the compressive strength, water absorption properties, and density. The compressive strength results have consistently indicated that peat mixtures possess lower compressive and higher absorption. The degree of reduction in strengths has been found to depend on the peat, cement proportions, and compaction pressure. The compressive strength of bricks when using Portland pulverized fuel ash cement (PFA) [10] was higher than the ordinary Portland cement (OPC) only when binding materials were 30%. Deboucha [11] reported that this brick is denser than aerated and lightweight concrete blocks and many other concrete masonry products, being about 15% to 20%. The water absorption and porosity of peat bricks decrease with the increase of cement content. Furthermore, for increasing cement content, the density increases.

Peat-added brick presented here eliminates the drawback of conventional fired bricks since no fuel is necessary for its production. This would contribute to solving energy and environment concerns simultaneously since proper addition of peat into brick results in better performance, as illustrated below. Even though its potential advantage, not much attention has been given in investigating structural performance of peat-added bricks. There is indeed limited number of researches on peat cementations products in the construction applications. Another thing is that in the previous studies a massive percentage of binding materials have greatly an effect on unit price of brick. In order to use structural application, other engineering parameters (such as flexural strength and spitting stress) are required to be investigated as a requirement with the relevant standards.

The work presented here focuses on the effect of peat addition as aggregates in cementation composites to develop the brick which is more cost effective minimizing the cement matrix. In this study, this work also attempts to investigate the attributes of this composite building material, which has a different percentage of peat and sand with cement, for the different application's purpose. Hence, this paper demonstrates the physicomechanical properties of the brick having different levels of peat as a replacement for sand aggregate. The test results that are illustrated from the investigation indicate that using minima cement with various peats for different structural application is potential as a lightweight low-cost building material.

## 2. Ingredients of Peat Added Brick

The innovative brick producing concept like peat added bricks has been studied to find out eco-friendly and cost-effective building component in the construction sector. Such composite brick uses locally available materials to meet the target. Materials used in this progression have been discussed along with their salient properties in the preceding sections.

### 2.1. Materials

Several raw materials have been used to manufacture the peat added brick. Brief descriptions of the materials are stated as follows.

- Peat soil used in this study was taken from Klang, Malaysia. It was dry enough to sieve and remove the coarse materials such as roots, stone, large fibers, and particles ranging in size from 2 mm to 0.075 mm.
- The siliceous sand materials are collected from the local market in Malaysia, the maximum which is 2 mm in size was used to increase solid matrix to the peat.
- The ordinary Portland cement (OPC).
- The hardness, sulfate content, and pH value of the supplied water are 3.7, 5.6 mg/L, and 6.2, respectively.

### 2.2. Characteristics of Peat Soil

Peat has high organic content over 75% and it is mainly of plant-rotten soil whose rate of accumulation is faster than the rate of decay [12, 13]. Its high organic and water content shows different mechanical properties and its consolidation settlements are time consuming even if moderate load is to be subjected [14, 15]. High water content, lower solid content, low pH values, and at the same time environment are also the factors affecting peat stabilization process [16, 17]. The physical and chemical components of peat change with time biologically and chemically. The properties of screen peat soils are presented in the Table 1. Huat et al. [12] reported that the liquid limit of peat soil is in the large range up to 500%. The higher value of bulk density is present in Table 1 due to subsiding, shrinkage, or mineralization.

### 2.3. Role of Cement

Cement and lime that are used in bricks as a source of reactive silica and alumina are given to silicate and aluminate hydrates and are responsible for the development of strength. The consolidation of the materials influence by the pozzolanic reaction in the binder and pozzolanic reaction depends on water content. Meukam et al. [18] indicated from their investigation that the compressive strength of stabilized laterite soil bricks varied between 2 MPa and 6 MPa with 8% cement content. According to Solomon-Ayeh [19] compressive strength of stabilized laterite soil bricks ranged between 2 MPa and 10 MPa with 3% to 10% cement content. In peat based bricks Deboucha and Hashim [8] state that, with the increasing cement content of 20% to 30%, the compressive strength increases by 40% and brick strength ranges from 2.9 to 7.6 MPa. They also reported that increasing the cement improved the dry density and decreased water absorption and porosity and it was found that the density in the bricks was increased from 5% to 7% and water absorption decreased from 68% to 14%. The most costly raw materials in this bricks are cement. The percentage of cement content affects the unit price of bricks, environment, and total costing of house construction. In this study, the authors like to take the cement as 20% and investigated relative composition of bricks, having different levels of peat as a replacement for sand aggregate, for the different application's purpose.

### 2.4. Effect of Sand Grain Size

To build strong stabilized peat, the grading of siliceous sand is very important because the void spaces contained by stabilized soil are reduced to a minimum when it is well packed with coarse grained sand filling the interstices with fine grained sand [20, 21]. The inclusion of the siliceous sand as filler produces no chemical reaction but enhances the strength of the stabilized peat by the binder due to increasing the number of soil particles available for the binder.

**Figure 1:**
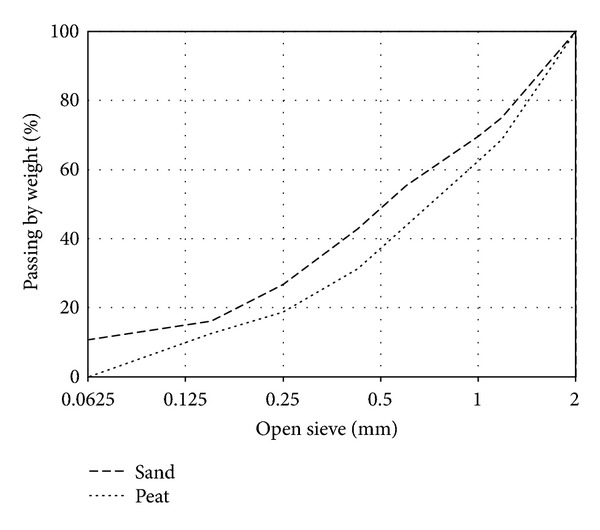
Grading curves of peat and sand.

The main purpose of cementation is to create soil water-resistance and to increase the compressive strength of structure. Ithnin [22] said that, theoretically, cement can stabilize all the soil. However, experimentally Adam and Agib [23] have shown that the increase of silt and clay content in the soil requires more cement to be added. To explain this reason, Hall and Allinson [24] confirmed this theory, “if soil content contains more finer particles than cement particles, this cannot be coated by cement.” So more cement is needed to ensure all particles being coated and it becomes uneconomical because it requires a substantial amount of cement than usual. The particle size of sand and peat greatly influences the percentage of cement content. The grading of screened peat soil and siliceous sand is presented in Figure 1.

## 3. Test Samples

Six various types of combinations have been prepared for the laboratory tests. In Table 2, the properties of fresh mixes are presented. The percentages of replacement between peat and siliceous sand are taken as weight replacements such as five percent replacement of peat soil means that five percent of the corresponding siliceous sand weight is exchanged by the peat and the corresponding specimen is presented as P-5. The peat soil is of less unit weight which means higher volume contents. The volumetric replacement corresponding to total volume is also shown in the Table 2.

The water percentage in the combinations is taken as a consultant to determine the effect of various sands and peat mixtures. The quantity of cement was taken as 20% of total peat plus sand weight for each combination.

To obtain the homogeneous mixing peat, sand and cement contents are placed in the electric mixer machine and mixed as dry in two to three minutes. It is seen that peat soil is evenly mixed within the mixes. Then water is added slowly into the mixer machine, while the mixer is rotating. An extra three minutes of mixing is performed. The mixtures are then fed into molds. The amount of water content has been increased with the increase of peat content. The applied water content values were defined according to standard proctor test [25]. The brick mold is fully filled with this fresh mixes having the proportions indicated in Table 2. Without any delay, the mix is pressed into the mold under pressure with a hydraulic jack machine. It was connected with a load cell and data-logger to control the pressure. After 3 to 5 minutes under pressure 7 MPa, the molded brick samples subsequently are air cured for 24 h. Later on, the mold is removed. When the formed brick was being demolded, any kind of damage was not observed. The brick sample is cured for duration of 28 days afterwards. In order to control the setting or hardening of cement and stop disintegrating, the brick sample was cured in humid environments for getting best results.

**Figure 2:**
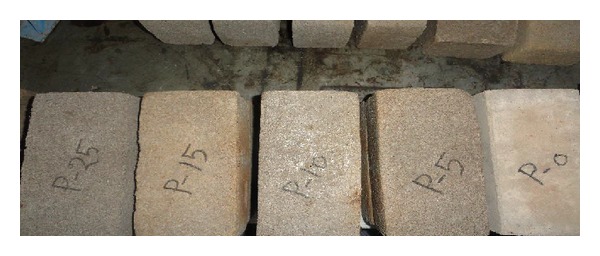
Appearance of brick samples.

The appearance of the peat added brick with various parts of peat content is shown in Figure 2. Following this procedure totally, 126 numbers of samples are prepared. Fifty-four bricks with a size of 100 mm × 70 mm × 220 mm are prepared for the test of flexural strength, splitting tensile strength, and UPV values. Additional 72 samples with a size of 70 mm × 70 mm × 70 mm have also been made for the test of compressive strength, unit weight, and water absorption.

## 4. Experimental Procedures

A series of tests considering various samples are undertaken according to ASTM C 67-03a, ASTM C 165-04, and ASTM C 67-02c to define the corresponding compressive strength, splitting strength, flexural strength, unit weight values, and water absorption values. The information with regard to these experimental procedures is represented here.

### 4.1. Water Absorption and Unit Weight

After finishing the 28 days curing period, the water absorption test is performed. The test samples are positioned into a ventilated oven at a constant temperature of 65°C. They are drawn out over a period of 48 h and weight is measured when it experiences room temperature. Afterward, samples are placed in the water tank and fully submersed for 48 h. The sample is taken out and kept for a while to drain out the surface water. At that juncture, using a damp cloth, the apparent saturated surface is removed and weighted immediately. This is the saturated weight of the sample. The water absorption is determined from this saturated weight and the dry weights. It is an important parameter for bricks. It indicates the permeability of bricks. The unit weight is calculated from their mass and overall volume of the sample.

### 4.2. Compressive Strength Test

The autocontrolled compression test machine is used to determine the compressive strengths of the sample. Maximum capability of compression of the universal testing machine is 800 KN. Both the applied load value together and compressive strength have been obtained from the autocompression test machine.

### 4.3. Flexural Strength and Ultrasonic Pulse Velocity (UPV) Tests

The flexural strength of the brick sample is determined through the three-point bending test. The width of sample is 100 mm, depth is 70 mm, and the supporting span is 160 mm. The ultrasonic pulse velocity (UPV) value is taken from the brick sample following [26] standard. This UPV value of a material is a function of its different sizes and volume fractions of aggregates and elastic modulus [27]. This value can be used to evaluate the materials uniformity and quality.

To determine the direct UPV values, the following methods are employed. A pulse transmitter is located on one side and a receiver on its direct opposite side of the brick sample. When the ultrasonic pulse transmitted through the brick length of 220 mm, its transit time is conveyed using a timing device. Then UPV of the sample is calculated using the relation: pulse velocity = (path length/transit time), where path length is the brick length.

### 4.4. Splitting Strength Test

The splitting strength of the peat added brick is done on the brick samples with a size of 100 mm × 70 mm × 220 mm. The compressive line load is applied through two 220 mm parallel steel edges. One is placed on the bottom of the sample and another at top. The splitting strength of brick is determined from the applied line load at which the tensile cracks form parallel to the brick edges.

## 5. Result and Discussion

**Figure 3:**
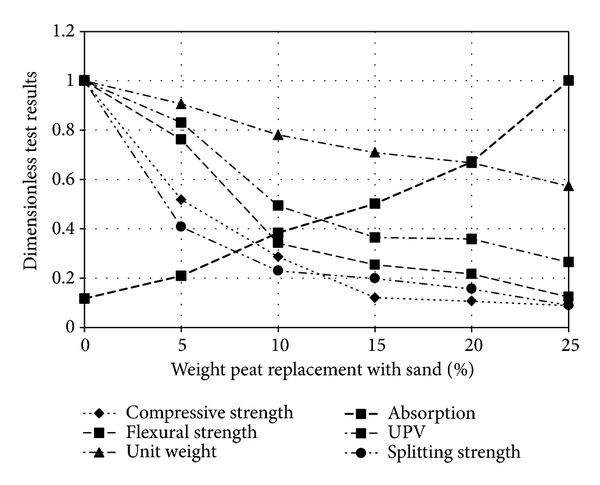
Dimensionless values for physicomechanical properties.

The test results from the experiment of peat added bricks have been shown in Figure 3 and Table 3. The water absorption and porosity with percentage of peat replacement with sand, compressive strength, and flexural strengths as well as comparative relationships among the compressive strength, UPV values, and the flexural strength are presented in Figures 5 and 7, respectively. Effect of curing time on the compressive strength of peat added brick is also evaluated. Detailed assessments have been discussed in the subsequent sections.

### 5.1. The Physicomechanical Properties

The physico-mechanical tests are performed to investigate whether the design samples satisfy the requirements as a construction material matching with the international standards. Figure 3 illustrates whole picture about the physicomechanical properties of sample obtained from the test series. The dimensionless ratios of parameters are plotted as a function of peat percentage. The dimensionless value for any parameter designates the ratio of the point value of the corresponding parameter and the maximum test result in this test series for the same parameter. The maximum result in each test series has been taken as a scaling parameter to the same parameter.

**Figure 4:**
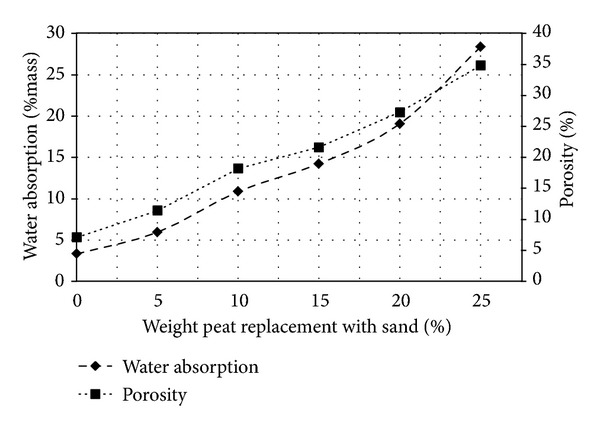
Water absorption and porosity with percentage of peat replacement with sand.

Figure 3 illustrates the maximum value of all parameters in this test series. The only exception of water absorption properties corresponds to that of the control mix specimen since unity values are found for 0% peat content. The test result obtained as of physical parameter tests is shown in Figure 4. Both the percentage of water absorption (percentage mass) and porosity of sample are monotonically increasing with increasing peat. On the other hand, the unit weight and UPV values decrease with peat percentage shown in Table 3. It is observed that increase in porosity results in decrease in UPV values and subsequently the unit weight reduces causing increase in water absorption.

Obtained test value represents qualitatively expected trend for lightweight building materials. In Figure 3 these trends illustrate more clearly where water absorption (percentage mass), UPV, and unit weight are considered as dimensionless values. It is seen that the dimensionless unit value of UPV and unit weight relates to control mix sample. On the other hand, in case of water absorption it relates to P-25 sample. This is for the reason that the maximum water absorption value 28.36% is obtained for the sample P-25 in the test series and thereby it is taken as scaling value.

### 5.2. The Physical Properties of the Samples

Peat presence highly influences the water absorption properties of bricks. It is known that peat has highly water content, and its liquid limit is above 150%. The 20% peat increases the water absorption 78%. However, it is clear that the water absorption comes below 20% with increase in peat up to 20%. The water absorption values that are obtained compared well with other similar materials and recommended maximum values for bricks. According to Ajam et al. [28], the water absorption values of PG fired bricks ranged from 15.84% to 19.67%. Kumar [29] and IS 1077–1992 [30] specify that the water absorption of ordinary burnt clay bricks should not be more than 20%. The quantitative evaluations of both parameters with the corresponding values in relevant international standards and past researches show that it satisfies up to P-20. Furthermore, the water absorptions match with the normally used bricks, such as clay bricks, 0 to 30%; concrete blocks, 4 to 25% [31].

Figure 3 indicates that unit weight values of the sample are inversely proportional to the percentage of peat. The unit weight value of control mix is 2150 Kg/m^3^ by considering it as an average unit weight of the block. The 20% peat decreases 33% of its weight and makes it 66% lighter. This reduction is particularly favorable showing the potential of peat-content bricks in the practice as a lightweight building material. As lightweight materials can reduce structural dead load, are easy to handle, reduce transportation costs, provide better thermal insulation, and increase the percentage of brick production per unit of raw material [32], there are also a negative relation between water absorption and unit weight, when increased water absorption and decreased unit weight. Kumar [29] reported that the increase in density of Fal.G. bricks was form 1172 Kg/m^3^ to 1230 Kg/m^3^, while water absorption decreased by about 19%. In these bricks, water absorption increase from 14% to 19%, while unit weight decreased from 1520 Kg/m^3^ to 1430 Kg/m^3^, and the trends are very high because peat absorbs comparatively high water than other soil.

Figure 4 illustrate the effect of peat in bricks porosity. Dhir and Jackson [33] reported that materials that have above 30% porosity are considered to be a high porosity. The 20% peat content bricks porosity 27.27 with less than 30 percent. All the bricks examined up to 20% peat content can therefore be considered to be of low porosity. According to Kerali [34], the decrease in compressive strength with increase in porosity can be party explained as the compressive strength of a block or brick is limited by brittle fracture. The peat and the sand matrix do not show any uneven surface and sudden brittle fracture even beyond the failure loads.

**Figure 5:**
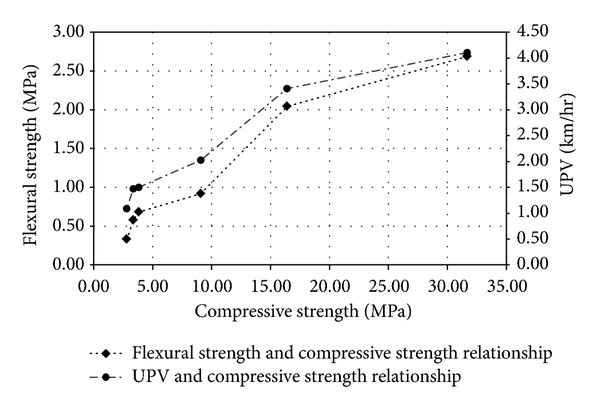
Comparative relationships of compressive strength, UPV values, and flexural strength.

The UPV is taken on the flexural strength brick samples having its 220 mm path length according to [26]. The comparative relationships among the compressive strength, UPV values, and flexural strength are shown in Figure 5. The UPV values are lower for the voids caused by peat. The reduction in the strength values causes the UPV to be decreased. Nondestructive UPV test results indicate that the compressive and flexural strength values of peat-add bricks may approximately be determined without a destructive testing, which gives a qualitative assessment of brick.

### 5.3. The Mechanical Properties of the Samples

Table 3 and Figure 3 demonstrate the mechanical test results of a series of samples. It can be revealed that the compressive strength, splitting strength, and flexural strength are found to be inversely proportionate with peat (percentage mass) replacement.

The compressive strength of bricks greatly is affected by peat that is illustrated in Figure 3. The presence of peat (P-5) made approximately half of its strength and then progressively decreased with peat percent. The compressive strength ranges for P-5 to P-25 are 16.40 to 2.80 MPa. In conventional, compressive strengths of compressed stabilized blocks were found to be no more than 4 MPa, and in some building authorities, authors recommend that compressive strength within the range of 3–5 MPa (nonload bearing) and 5–10 MPa (load bearing) may be sufficient [32, 35, 36]. Some also recommended that minimum values of compressive strength are 1.2 MPa and 2.8 MPa [9, 37]. Adam and Agib [23] in their studies compared the practical value of compressive strength of some common bricks and block. They found that the compressive strength of compressed stabilized earth blocks and light weight concrete blocks was within the range 1–40 MPa and 2–20 MPa, respectively. According to TS 705 [38], the minimum compressive strength of masonry units in both cases of nonload bearing and loads bearing is comparatively much lower: correspondingly 2.5 and 5.0 MPa. For the British Standard, the minimum requirement for precast concrete masonry units and fired clay blocks is 2.8 MPa and for bricks is 5.2 MPa, where the building is light load. In IS 1077–1992 [39], bricks are classified into eleven groups, based on their average compressive strength. According to this classification, the minimum compressive strength of brick should be 3.5 MPa. Hence, it shows that sample P-20 can be used in nonload bearing and load bearing masonry units for low cost and lightweight building construction. According to ASTM C 129 [40], the sample maximum P-15 can be used for the nonload bearing masonry units, where the minimum value of ASTM C 129 [40] is 3.50 MPa. The samples P-10 satisfy the minimum value 7.0 MPa for the building material to be used in the structural applications, as described in BS 6073 [41] and also close to ASTM C 129 [40] for load bearing.

**Figure 6:**
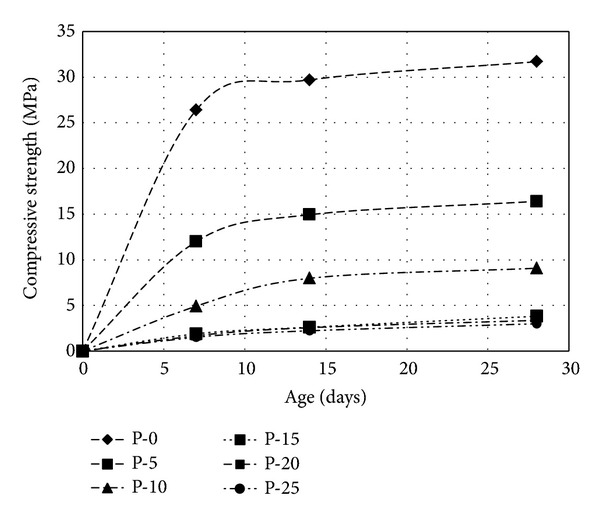
Effect of curing time on the compressive strength.

The hydration process of cement is a quite long reaction, will continuously modify through days, months, and even years, and increases its mechanical strength [42]. In Figure 6 the strength of brick with age and effect of curing are illustrated.

It is seen that control sample gains more than 80% strength within first 7 days, but in presence of peat, it is around 50%. The P-5 to P-25 sample shows the similar trend of increasing strength with days.

**Figure 7:**
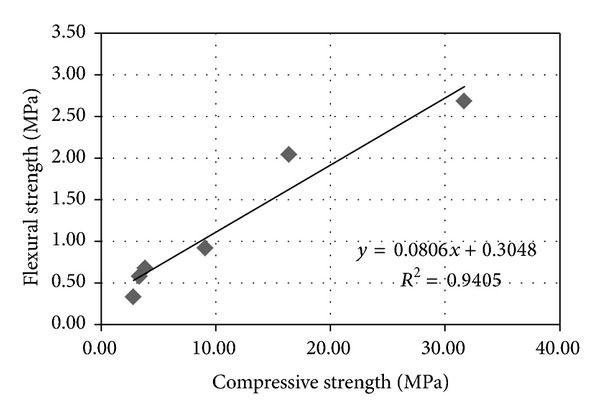
Correlation between the compressive strength and the flexural strengths.

Figure 7 represents a linear relationship between the two parameters of bricks. The splitting stress at which the brick sample may crack is a form of tension failure (Table 3). Minimum flexural strength described in BS 6073 [41] that is 0.65 MPa for building materials can be used in structural applications. The flexural strength of P-20 samples (0.58 ± 0.05 MPa) and bricks with maximum 15% peat replacements satisfy the standard BS6073. Hence, these peat added bricks could to be used in structural applications.

## 6. Conclusion

The feasibility of producing peat-added bricks has been shown technically in this study. For this intend, the physicomechanical properties of peat bricks are examined. The result illustrates that maximum 20% weight replacement or 44% volumetric replacement of peat can satisfy the requirement of relevant international standards. Based on the experimental investigation covered in this paper, further conclusions are drawn as follows.

- Compressive strength and flexural strength of brick sample P-15 satisfy the minimum requirement for load-bearing masonry unit that can be utilized in the structural applications and nonload-bearing masonry units bricks sample P-20 that can be applied.
- Although 25% replacement with sand by peat achieves the minimum compressive strength of available compressed stabilized earth bricks, water absorption and porosity are markedly affected its durability.
- The water absorption, density, and porosity of bricks are greatly influenced by peat than cement percentage.
- The higher void and large coarse soil particles in bricks can create flaws and weaker the bricks. The siliceous sand and peat soil fraction having a particle size not more than 2 mm and for increasing sand matrix peat soil fraction greater than zero are used, which provides comparatively better results. The uneven surface and sudden brittle fracture are not exhibited even at ultimate loads.
- The physicomechanical properties of bricks at different peat level presented in this study illustrate that peat bricks with 20% cement content have potential to be used as lightweight low cost masonry unit.

This study may offer significant savings in not only materials and energy saving, but also a unique kind of environment friendly building material feature and an economic alternative to the conventional bricks. The construction of houses using local materials, in the developed countries, became marginal and it was abandoned because it is complex to standardize the composition of materials as it varies locally. Therefore, detailed further study is needed to figure out a complete guideline for local peat soil of different region in Malaysia to prepare eco-friendly and cost-effective peat added bricks.

## Figures and Tables

**Table 1:** Properties of screened peat soil.

Properties	Value
Bulk density *(*γ*b)*	1.1 Mg/m^3^
Dry density *(*γ*d)*	0.194 Mg/m^3^
Fiber content	80%
Specific gravity (Gs)	1.48
Void ratio (*e*)	7.5
Classification/Von Post	H4
Liquid limit	165.2%
Plastic limit	125.10%
Plasticity index	40.1%
Linear shrinkage	5.6%
pH	3.6

**Table 2:** Mixing composition of one brick sample.

Mix design	Cement (g)	Water (g)	Sand (g)	Peat soil (g)	Total (g)	Optimum moisture content (%)	Percentage of peat volume (%)
Control mix	991	416	2313	0	3720	18	0%
P-05	898	395	1946	150	3389	19	13%
P-10	773	356	1547	258	2934	20	28%
P-15	703	337	1288	351	2679	21	37%
P-20	661	331	1102	441	2535	22	44%
P-25	568	284	852	473	2177	22	54%

**Table 3:** Experimental values of the peat brick samples.

Sample	Compressive strength (MPa)	Unit weight (kg/m^3^)	Flexural strength (MPa)	Splitting strength (MPa)	UPV value (Km/h)
Control mix	31.70	2145.33	2.69 ± 0.20	3.35 ± 0.01	4.10 ± 0.13
P-5	16.40	1943.91	2.04 ± 0.18	0.85 ± 0.03	3.41 ± 0.04
P-10	9.08	1674.11	0.92 ± 0.03	0.48 ± 0.01	2.03 ± 0.05
P-15	3.82	1521.00	0.68 ± 0.04	0.41 ± 0.01	1.50 ± 0.02
P-20	3.37	1431.34	0.58 ± 0.05	0.32 ± 0.02	1.47 ± 0.11
P-25	2.80	1229.76	0.33 ± 0.04	0.19 ± 0.04	1.09 ± 0.01
